# Long-Term Survival of a Patient With Anaplastic Thyroid Carcinoma Treated With Hypofractionated Radiotherapy: A Case Report

**DOI:** 10.7759/cureus.56689

**Published:** 2024-03-22

**Authors:** Mitsuki Tanaka, Yoshiomi Hatayama, Hideo Kawaguchi, Ichitaro Fujioka, Masahiro Aoki

**Affiliations:** 1 Department of Radiation Oncology, Hirosaki University Graduate School of Medicine, Hirosaki, JPN

**Keywords:** hypofractionated radiotherapy, external beam radiotherapy (ebrt), radiotherapy (rt), thyroid cancer, anaplastic thyroid carcinoma

## Abstract

Anaplastic thyroid carcinoma, a rare type of primary thyroid cancer, is one of the most aggressive neoplasms with a poor prognosis. Many cases are in the advanced stage at the time of the initial visit, and curative treatment is impossible. Because of the highly radioresistant nature of anaplastic thyroid carcinoma, this condition cannot be properly controlled with conventional radiotherapy.

Herein, we report the case of a patient with anaplastic thyroid carcinoma who underwent hypofractionated radiotherapy, attained a complete response, and is still alive more than 10 years after treatment with no evidence of disease. To overcome the high radioresistance of anaplastic thyroid carcinoma, we administered 50 Gy in 10 fractions three times a week. Furthermore, we administered paclitaxel and carboplatin sequentially before and after radiotherapy. Consequently, the patient completed treatment and reached a complete response. He is still alive more than 10 years after treatment with no evidence of disease or severe adverse events. Hypofractionated radiation therapy may provide good control of locally advanced anaplastic thyroid carcinoma.

## Introduction

Anaplastic thyroid carcinoma, whose prognosis is poor, is one of the most aggressive neoplasms. The one-year overall survival and median survival time have been reported to be 20% and only five months, respectively [1]. Many cases of the disease are diagnosed at an advanced stage, which makes curative treatment almost impossible. Because I-131 therapy is ineffective, multidisciplinary treatments such as surgery, external beam radiotherapy, and chemotherapy are being performed; however, there is currently no evidence-based standard treatment for this condition. Surgery and postoperative chemoradiotherapy are recommended in resectable cases. For unresectable cases, chemoradiotherapy for local control is recommended, and if it becomes resectable after tumor shrinkage, surgery will be performed. Palliative care is recommended for patients with distant metastases due to the poor prognosis of the condition [2].

Herein, we present the case of a 50-year-old Japanese male with locally advanced anaplastic thyroid carcinoma who was treated with hypofractionated radiotherapy and sequential chemotherapy. He attained a complete response and is still alive more than 10 years after treatment with no evidence of disease.

## Case presentation

**Figure 1:**
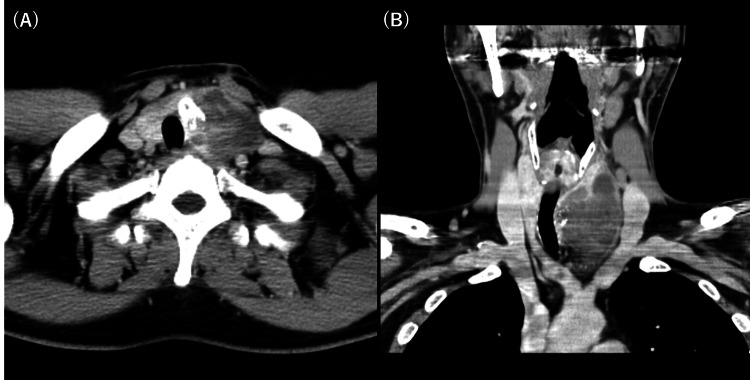
(A) Axial image; Computed tomography (CT) revealed a tumor in the left lobe of the thyroid gland, and the tumor invaded the adipose tissue around the thyroid gland on axial image. (B) Coronal image; the size of the tumor was 7 cm.

**Figure 2:**
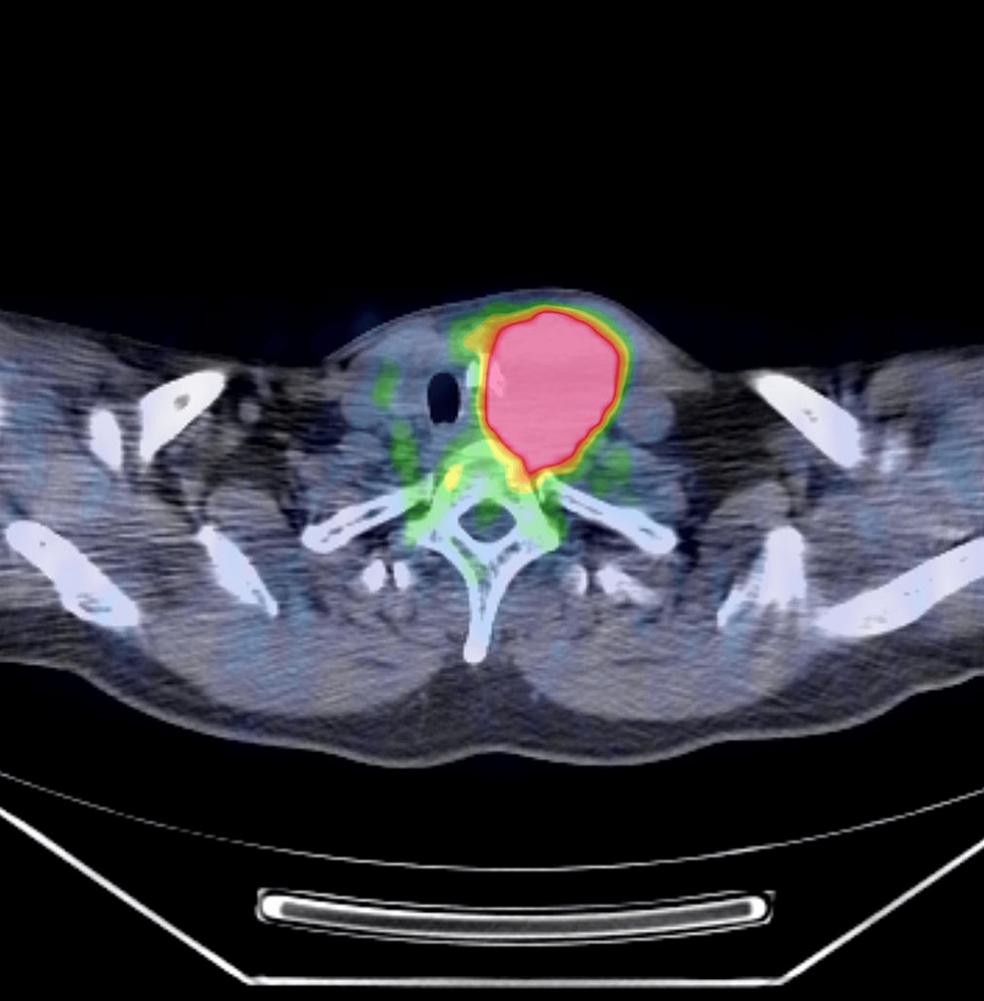
Positron emission tomography-computed tomography (PET-CT) revealed strong accumulation of 18F-fluorodeoxyglucose (18F-FDG) in the tumor, and no enlargement of regional nodes or distant metastasis was revealed.

A 50-year-old Japanese man noticed a left cervical mass which increased in size weekly and experienced dysphagia in 2012. One month after the onset of his symptoms, he visited the hospital. Computed tomography (CT) revealed a tumor measuring 7 cm in diameter in the left lobe of his thyroid gland, and this tumor invaded the adipose tissue around the gland (Figure 1). Positron emission tomography-computed tomography (PET-CT) revealed strong ^18^F-fluorodeoxyglucose (^18^F-FDG) accumulation in the tumor, and no enlargement of regional nodes or distant metastasis was observed (Figure 2).

**Figure 3:**
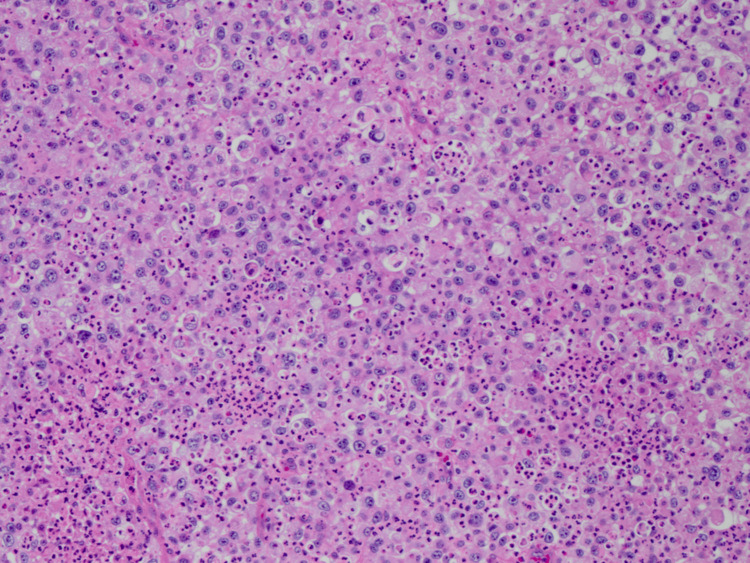
The tumor comprises large atypical cells with extensive eosinophilic cytoplasm. There was no normal thyroid tissue.

An incisional biopsy was performed. The tumor, alongside the intra-tumoral abscess, measured 7 × 5 cm in size. The tumor, which had extensive adhesions, invaded the left internal carotid artery. The tumor comprises large atypical cells with extensive eosinophilic cytoplasm (Figure 3). Irregular necrosis was also observed in the tumor. There was no normal thyroid tissue. The number of neutrophils inside and around atypical cells was significantly increased, and neutrophilic infiltration of the tumor cell cytoplasm (emperipolesis) can be observed. The tumor cells tested negative for CD45, CD20, CD79a, CD3, CD68, CD163, CEA, EMA, MUM-1, and ALK, which are markers of malignant lymphoma, lymphoblastic leukemia, and histiocytosis. The tumor cells tested negative for CK7 and CK20 and negative for TTF-1, SP-A, and MapsinA, which are markers of lung cancer. They also tested negative for CDX2, which is a marker of gastrointestinal cancer. The tumor cells tested negative for HMB45 and melanA, which are markers of melanoma, and negative for synaptophysin and chromogranin, which are markers of neuroendocrine tumors. The tumor cells tested partially positive for cytokeratin AE1/AE3 and CD138; therefore, they were highly suspected to be carcinoma. The tumor’s histology was atypical for a tumor originating in the thyroid gland, and tumor cells tested negative for thyroglobulin and diffusely positive for G-CSF. Based on these results, the tumor was identified as anaplastic thyroid carcinoma. Finally, he was diagnosed with clinical T3bN0M0, stage IVB anaplastic thyroid carcinoma.

**Figure 4:**
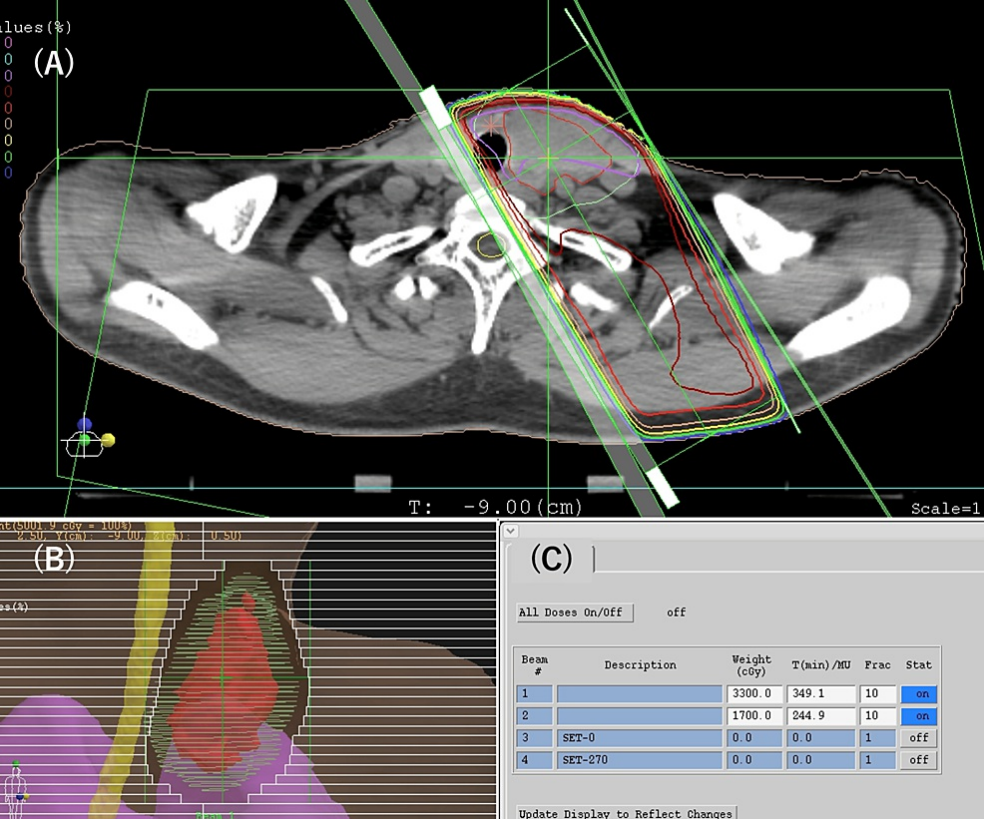
(A) Dose distribution map; the gross tumor volume (GTV), which was defined as the volume of the primary thyroid tumor, is shown in red. The clinical target volume (CTV) was defined as GTV plus 0.5 cm margin in all directions, and the planning target volume (PTV) was defined as CTV plus 0.5 cm margin in all directions (it is shown in green). A total dose of 50 Gy at the isocenter of the PTV was given in 10 fractions three times a week. The GTV and most of the PTV are surrounded by the 100% isodose line. (B) Beams eye view; we completely blocked the spinal cord. (C) Beams weight

Initially, one course of chemotherapy with paclitaxel (200 mg/m^2^) and carboplatin (5AUC) was administered. Subsequently, hypofractionated radiotherapy was performed. We used a three-dimensional planning procedure. The gross tumor volume (GTV) was defined as the volume of the primary thyroid tumor, the clinical target volume (CTV) was defined as GTV plus a 0.5-cm margin in all directions, and the planning target volume (PTV) was defined as CTV plus a 0.5-cm margin in all directions. The prophylactic lymph node area was not irradiated. We used 10- and 6-megavolt X-rays and horizontally opposing pair fields (Figure 4). A total dose of 50 Gy at the isocenter of PTV was administered in 10 fractions thrice weekly. No chemotherapy was performed during radiotherapy. After radiotherapy, nine courses of the same regimen of chemotherapy were administered every three weeks.

**Figure 5:**
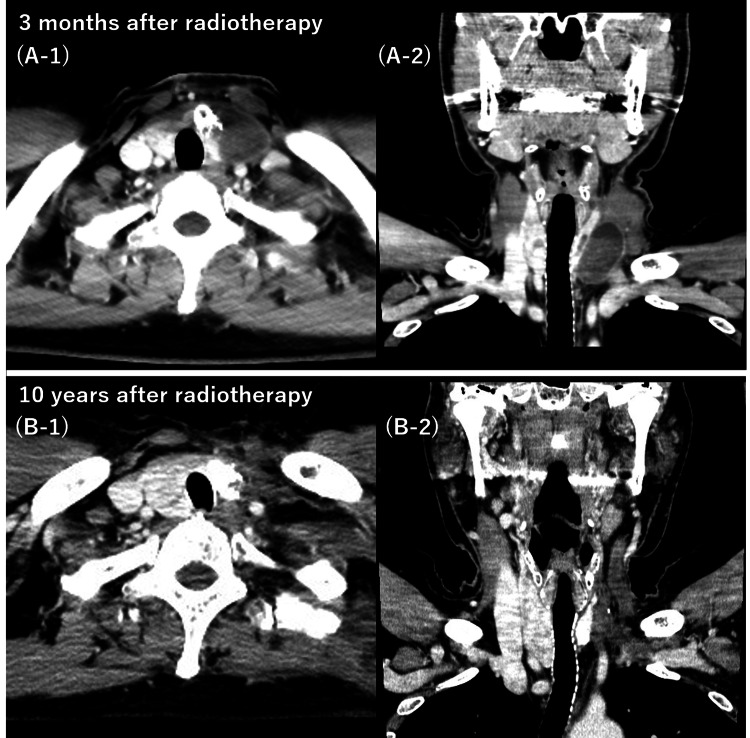
Six months after radiotherapy, it shrunk further and was hidden and this condition continued until 10 years after the end of radiotherapy. (A-1) Three months after radiotherapy, axial image; the tumor had shrunk slightly. (A-2) Three months after radiotherapy, coronal image (B-1) 10 years after radiotherapy, axial image; the tumor shrunk further and was hidden and this condition continued until 10 years after the end of radiotherapy. (B-2) 10 years after radiotherapy, coronal image

Immediately after radiotherapy, the tumor size showed almost no change on CT and palpation; however, three months after radiotherapy, the tumor had shrunk slightly. Six months after radiotherapy, it shrunk further and was hidden and this condition continued until 10 years after the end of radiotherapy. The patient is still alive more than 10 years after treatment with no evidence of disease (Figure 5).

Grade 2 radiation dermatitis and grade 2 radiation esophagitis were observed as acute adverse events due to radiotherapy. Grade 3 leukocytopenia, grade 4 neutropenia, grade 1 anemia, and grade 1 thrombocytopenia were observed as acute adverse events due to chemotherapy. Grade 2 soft tissue fibrosis and grade 2 left brachial plexopathy were observed as late adverse events due to radiotherapy.

## Discussion

Although anaplastic thyroid carcinoma accounts for less than 2% of all cases of thyroid cancer, it accounts for 14%-39% of thyroid cancer deaths [3,4]. Kebebew et al. reported that intrathyroidal tumors, extrathyroidal tumors and/or lymph node invasion, and distant metastasis occurred in 8%, 38%, and 43% of cases, respectively [5]. Therefore, many cases of anaplastic thyroid carcinoma are radically unresectable at the time of the initial hospital visit, and chemoradiotherapy plays an important role in the treatment of anaplastic thyroid carcinoma. However, because of the poor prognosis and rarity of anaplastic thyroid carcinoma, no evidence-based standard therapy has been established so far [2].

Molecular targeted therapy has recently been approved for inoperable cases; however, complete remission is exceptional, and adverse events occur frequently [6]; therefore, administration should be carefully decided. Although there was no distant metastasis, this case was inoperable due to the direct invasion of the left internal carotid artery. Therefore, it was planned to perform radiotherapy as local therapy.

There is no other report of a patient with anaplastic thyroid carcinoma who survived for a long time with radiation therapy alone like the patient in our study. Anaplastic thyroid carcinoma is considered to have low inherent radiosensitivity and a very short potential doubling time, which makes it highly radioresistant [7]. To overcome these obstacles, we reduced the overall treatment time by increasing the fraction size, and the total dose was equivalent to a 2 Gy fraction (EQD2) ≥50 Gy. A dose of 5 Gy per fraction was selected for this treatment because hypofractionated radiotherapy of 4-7 Gy per fraction has been safely performed for head and neck SCC, melanoma, and facial skin cancer [8-11]. However, too-large doses per week cause severe acute adverse events [12]; therefore, we irradiated thrice weekly as a preventive measure. Patients with anaplastic thyroid carcinoma who received EQD2 ≥50 Gy have longer overall survival periods than those who received EQD2 of <50 Gy [13,14]. Our 50 Gy in ten fractions corresponds to a dose of almost 60 Gy in EQD2, so it is expected that the overall survival will be relatively long. Because of the possibility of a severe adverse event, chemotherapy was not used alongside radiotherapy. This treatment strategy led to a good prognosis, and the adverse events incurred were acceptable.

Clinical results of conventional fractionation radiotherapy, accelerated hyper-fractionated radiotherapy, and hypofractionated radiotherapy for anaplastic thyroid carcinoma are reported in Table 1. The median overall survival is less than one year for all patients; however, local control is relatively good at 74%-85% by the time of death. Accelerated hyper-fractionated radiotherapy and hypofractionated radiotherapy have been used to shorten the overall treatment duration and improve treatment results. However, accelerated hyper-fractionated radiotherapy is poorly tolerated, with high rates of grade 3 or 4 acute adverse events, including radiation dermatitis (56%), dysphagia (74%), and esophagitis (79%) [15]. On the other hand, in hypofractionated radiotherapy, grade 3 acute adverse events have been observed in 41% of patients but grade 4 events have not been observed, and it seems to be more acceptable than accelerated hyper-fractionated radiotherapy [16]. In this study, we demonstrated that 50 Gy in 10 fractions of thrice-weekly radiotherapy for anaplastic thyroid carcinoma resulted in long-term survival. Other studies on the treatment of anaplastic thyroid carcinoma using the same radiotherapy schedule that was used in this study reported good local control rates (74%-90%) are obtained and adverse events are acceptable [13,17]. The longest overall survival for patients treated using this radiation therapy schedule is 17 months. There is also a report that 21.6% of patients survived for more than five years with different radiotherapy schedules [14]; however, in all cases, these were patients who underwent thyroidectomy before or after radiotherapy. To the best of our knowledge, there are no previous reports of patients surviving long-term with hypofractionated radiotherapy and sequential chemotherapy alone.

**Table 1:** Major radiation series comparing outcomes of anaplastic thyroid carcinoma.

Author	Median radiation dose (range)	Frequently used radiation doses and fractions	Resection	Chemotherapy	Grade3 or higher toxicity	Local control	Median survival time
Takahashi et al [13].	50 Gy (5-60)	50 Gy in three times weekly fractions of 5 Gy	26%	42%, concurrent	Skin toxicity (Grade3; 5%), Dysphagia (Grade3; 26%), Esophagitis (Grade3; 5%), Trachea necrosis (Grade4; 5%)	74% (at the time of death)	3.4 months
Takahashi et al [13].	55.5 Gy (6-70)	60 Gy in a daily fraction of 2-3 Gy	50%	50%, concurrent	Skin toxicity (Grade3; 7%), Dysphagia (Grade3; 36%), Esophagitis (Grade3; 14%)	50%	5.2 months
Dumke et al [14].	50 Gy (6–60.4)	Single dose is unknown. Daily (87.5%), every other day (5%), twice daily (5%).	80%	15%	Skin toxicity (Grade3; 13%), Dysphagia (Grade3; 25%, Grade4; 3%)	Unknown	5 months
Dandekar et al [15].	57 Gy (7.6-60.8)	60.8 Gy in twice daily fractions of 1.8 and 2 Gy	32%	Unknown	Erythema (Grade3; 38%, Grade4; 18%), Desquamation (Grade3; 12%, Grade4; 9%), Dysphagia (Grade3; 30%, Grade4; 44%), Esophagitis (Grade3; 30%, Grade4; 47%)	85% (at the time of death or last follow up)	70 days
Stavas et al [16].	54 Gy (40-62.5)	54 Gy in a daily fraction of 3Gy	89%	88%, concurrent	Skin toxicity (Grade3; 24%), Dysphagia (Grade3; 28%), Esophagitis (Grade3; 18%)	82% (at the time of death)	9.3 months
Jingu et al [17].	50 Gy (40-50)	50 Gy in three times weekly fractions of 5 Gy	Unknown	50%	Unknown	90% (1 year)	About 3 months

In our case, there were no lymph node metastases or distant metastases, and the irradiation target was limited to the thyroid gland; therefore, treatment with 50 Gy in 10 fractions of thrice-weekly radiotherapy was possible. For anaplastic thyroid carcinoma in which the tumor is localized only in the thyroid gland, this hypofractionated radiotherapy schedule may provide long-term survival even in inoperable patients. This radiotherapy schedule has the advantage that patients can rapidly switch to it from treatments such as chemotherapy. This patient completed hypofractionated radiotherapy without severe acute adverse events and is still alive more than 10 years after treatment with no evidence of disease or severe late adverse events. This hypofractionated radiotherapy schedule is considered a safe and effective treatment option for anaplastic thyroid carcinoma.

## Conclusions

In this paper, we reported the case of a 50-year-old Japanese male with locally advanced anaplastic thyroid carcinoma who was treated with hypofractionated radiotherapy and sequential chemotherapy. With 50 Gy in 10 fractions of thrice-weekly radiotherapy and sequential chemotherapy, the patient attained a complete response and is still alive more than 10 years after treatment with no evidence of disease or severe adverse events. We believe that this case is extremely rare and will be of global interest, and we are proud to report the good control of locally advanced anaplastic thyroid carcinoma procured by hypofractionated radiotherapy.
